# The Use of a Micro Near Infrared Portable Instrument to Predict Bioactive Compounds in a Wild Harvested Fruit—Kakadu Plum (*Terminalia ferdinandiana*)

**DOI:** 10.3390/s21041413

**Published:** 2021-02-18

**Authors:** Eshetu Bobasa, Anh Dao T. Phan, Michael Netzel, Heather E. Smyth, Yasmina Sultanbawa, Daniel Cozzolino

**Affiliations:** 1 ARC Industrial Transformation Training Centre for Uniquely Australian Foods, Queensland Alliance for Agriculture and Food Innovation, The University of Queensland, 39 Kessels Rd, Coopers Plains, Brisbane, QLD 4108, Australia; e.bobasa@uq.edu.au (E.B.); a.phan1@uq.edu.au (A.D.T.P.); m.netzel@uq.edu.au (M.N.); h.smyth@uq.edu.au (H.E.S.); y.sultanbawa@uq.edu.au (Y.S.); 2 Centre for Nutrition and Food Sciences, Queensland Alliance for Agriculture and Food Innovation, The University of Queensland, St. Lucia, Brisbane, QLD 4072, Australia

**Keywords:** near infrared, vitamin C, ellagic acid, wild harvest, Kakadu plum

## Abstract

Kakadu plum (KP; *Terminalia ferdinandiana* Exell, Combretaceae) is an emergent indigenous fruit originating from Northern Australia, with valuable health and nutritional characteristics and properties (e.g., high levels of vitamin C and ellagic acid). In recent years, the utilization of handheld NIR instruments has allowed for the in situ quantification of a wide range of bioactive compounds in fruit and vegetables. The objective of this study was to evaluate the ability of a handheld NIR spectrophotometer to measure vitamin C and ellagic acid in wild harvested KP fruit samples. Whole and pureed fruit samples were collected from two locations in the Kimberley region (Western Australia, Australia) and were analysed using both reference and NIR methods. The standard error in cross validation (SECV) and the residual predictive deviation (RPD) values were 1.81% dry matter (DM) with an RPD of 2.1, and 3.8 mg g^−1^ DM with an RPD of 1.9 for the prediction of vitamin C and ellagic acid, respectively, in whole KP fruit. The SECV and RPD values were 1.73% DM with an RPD of 2.2, and 5.6 mg g^−1^ DM with an RPD of 1.3 for the prediction of vitamin C and ellagic acid, respectively, in powdered KP samples. The results of this study demonstrated the ability of a handheld NIR instrument to predict vitamin C and ellagic acid in whole and pureed KP fruit samples. Although the RPD values obtained were not considered adequate to quantify these bioactive compounds (e.g., analytical quantification), this technique can be used as a rapid tool to screen vitamin C in KP fruit samples for high and low quality vitamin C.

## 1. Introduction

Kakadu plum (KP; *Terminalia ferdinandiana* Exell, Combretaceae) is an emerging indigenous fruit originating from Northern Australia, with valuable health and nutritional characteristics and properties such as high levels of vitamin C, ellagic acid, and other polyphenolic compounds [1,2,3,4,5,6]. Kakadu plum is the most common name for this fruit, and it is found from the Kimberley (Western Australia) to Darwin (Northern Territory) and Queensland regions [1,2,3,4,5,6]. Commercial harvesting of KP fruit started in the late 1990s. While the vast majority of production is from wild harvested fruit [1,2,3,4,5,6], some commercial orchards can be found in Australia. Like many wild-harvested native foods, weather conditions, including drought, bushfires, and cyclones, might have an impact on the volume of fruit available, so production is highly variable from year to year [1,2,3,4,5,6]. The main harvest time is January, although some trees have multiple flowerings and can produce fruit up until July, depending on the region. The production of this fruit is estimated to average 15–17 tonnes per annum [1,2,3,4,5,6]. Although the KP is commercialised as whole fruit, it can be processed as a pureed or dehydrated powder (e.g., freeze dried) [7]. The dehydrated powder is used as a functional food ingredient in order to add value to a wide range of different food products (e.g., yogurts and ice creams), a common practice in the food industry [1,2,3,4,5,6,7]. It is well recognised that the health benefits of native plants are attributed to the content of antioxidant compounds such as natural ascorbic acid (vitamin C) and polyphenols, including gallic and ellagic acids [4,5]. These antioxidants have become very important in human health and nutrition, motivating the rapidly expanding search for plant sources containing these compounds in the wild (e.g., native plants). Kakadu plant materials (e.g., fruit and leaves) have high quantities of ellagic acid, together with the bioactive forms of vitamin C (ascorbic acid), making this plant very attractive as a source of natural antioxidants [4,5,6].

In recent years, applications based on the use of vibrational spectroscopy (near infrared, mid infrared, and Raman) have been utilised to quantify and monitor the composition and nutritional value in a wide range of plant and fruit materials [8,9,10,11,12,13]. In particular, the use of near infrared (NIR) spectroscopy has demonstrated that it can be a versatile tool to analyse different types of samples and conditions [8,9,10,11,12,13]. These recent developments in portable and handheld instrumentation have opened a new window for utilising these types of instruments to analyse and monitor the composition of fruit and vegetables [8,9,10,11,12,13,14]. In this context, the utilization of handheld instrumentation is allowing the quantification of antioxidants and bioactive compounds in native or wild harvest fruit samples like KP fruit.

Therefore, the objective of this study was to evaluate the ability of a handheld NIR instrument combined with chemometrics to measure vitamin C and ellagic acid concentrations in KP fruit samples.

## 2. Materials and Methods

### 2.1. Samples

Kakadu plum fruit samples were wild harvested in January 2020 from two different locations in the Kimberley region (Western Australia, Australia). Ten KP trees from each site were randomly selected for harvesting (approximately 50–100 fruit per tree). The samples were stored and transported to the laboratory under refrigerated conditions and then immediately stored at −80 °C for further analysis. The frozen fruit samples were thawed at room temperature (20 °C) before the NIR and reference analyses. After NIR scanning, the fruit samples were blended into a puree using a mortar and pestle. Consequently, the obtained pureed samples were analysed as a puree using the same NIR spectrophotometer as described in the section below (infrared spectroscopic measurements). Following the NIR analysis, the pureed samples were lyophilized (Lindner and May Ltd., Windsor, QLD, Australia) and finely ground using a Retsch MM301 cryomill (Retsch GmbH, Haan, Germany) in order to provide a uniform powder for the determination of vitamin C and ellagic acid. After all of the fruit samples underwent NIR scanning, representative samples were selected using principal component analysis in order to be utilised for further reference analysis and calibration development.

### 2.2. Infrared Spectroscopic Measurements

The NIR spectra of either whole (*n* = 60) or pureed (*n* = 60) KP fruit samples were collected using a portable NIR spectrophotometer (Micro-NIR 1700, Viavi, Milpitas, CA, USA) operating in a 950–1600 nm wavelength range, with a spectral resolution of 10 nm with no moving parts (Viavi Solutions, 2015, Milipitas, CA, USA). The NIR instrument was connected through a USB interface to a notebook computer running proprietary software (MicroNIR Prov 3.1, Viavi, Milpitas, CA, USA) for the acquisition of the diffuse reflectance spectra of the samples (Viavi Solutions, 2015, Milipitas, CA, USA). The controlling parameters for the spectral data acquisition were set at 50 min integration time and with an average of 50 scans (MicroNIR Prov 3.1, Viavi, Milpitas, CA, USA). The reference spectra for the absorbance/reflectance calculations were collected using Spectralon^®^ after the consecutive scanning of 10 samples. 

### 2.3. Determination of Ellagic Acid

The extraction and analysis of ellagic acid (EA) were conducted according to the method previously reported by Williams and collaborators, with some modifications [5,6]. Briefly, 100 mg of powdered samples were extracted with 80% methanol containing 0.01N HCl using a vortex, followed by sonication for 10 min. The free EA released in the supernatant (referred to as extract A) was collected after being centrifuged (3220× *g*, 5 min at 20 °C; Eppendorf Centrifuge 5810 R, Hamburg Germany), whereas the residues were extensively extracted with absolute methanol in order to completely release the remaining free EA (extract B). 

In order to measure the EA existing under bound form (e.g., ellagitannins), hydrolysis was conducted following the method reported by Williams and collaborators [5,6]. The obtained extract A was added into a 5 mL Reacti-Therm vial (Fisher Scientific, Bellefonte, PA, USA) and subjected to overnight hydrolysis at 90 °C using 2N HCl. The EA released after hydrolysis was dissolved in methanol (referred to as extract C) before the UPLC-PDA analysis.

EA in three different extracts was analysed using a Waters AcquityTM UPLC-PDA System (Waters, Milford, MA, USA). The compound was separated on a Waters BEH Shield RP C18 column (100 × 2.1 mm i.d; 1.7 µm) maintained at 35 °C. The mobile phases included 0.1% formic acid (FA) in Milli-Q water (A) and 0.1% FA in methanol (B). The flow rate was 0.3 mL/min, with the following gradient elution for B: 35% B isocratic conditions for 5 min, 50% B for 10 min, and 100% B for 15 min. The contents of free EA (extracts A and B) and total free and bound EA (extracts B and C) were quantified using an external calibration curve of ellagic acid acquired at 254 nm [5,6].

### 2.4. Determination of Vitamin C

The extraction and analysis of vitamin C in the powder samples were conducted following a method previously described elsewhere [15]. Briefly, 100 mg of powdered KP samples were extracted with 3% meta-phosphoric acid containing 8% acetic acid and 1 mL ethylenediaminetetraacetic acid (EDTA). The reduction of dehydroascorbic acid (DHAA), which was also present in the extracts/samples, to ascorbic acid (L-AA) was performed [15,16]. The total vitamin C (L-AA + DHAA) was determined using a Waters UPLC-PDA system and a Waters HSS-T3 column (150 × 2.1 mm i.d; 1.8 μm; 25 °C), with water with 0.1% formic acid as the mobile phase (0.3 mL/min) under isocratic elution. Vitamin C was quantified using an external calibration curve of ascorbic acid acquired at 245 nm [15].

### 2.5. Data Analysis

The NIR spectra were pre-processed (second derivative, second order polynomial, 21 smoothing points) using The Unscrambler software (version 11, CAMO, Oslo, Norway) [17]. A principal component analysis was conducted using The Unscrambler software, after a second derivative with cross validation (full cross validation) [18]. Partial least squares regression (PLS) was used to relate the NIR spectra with the content of vitamin C and ellagic acid in the KP fruit samples analysed. To evaluate the performance of the PLS models, validations were performed on two different datasets. For the purpose of this study, the original dataset was split into two subsets of 70% (e.g., calibration) and 30% (e.g., validation), using the Kennard-Stone algorithm [19]. Thus, 40 uniformly distributed samples were selected and used in the calibration, while 20 samples were used for validation. By performing data partitioning, knowledge of the training dataset did not affect the test dataset, and the predictive power of the created model subsequently increased. Leave-one-out cross-validation was applied on the calibration set for internal validation, and the test set was used to externally validate the generated models. The coefficient of determination (R^2^), the standard error in cross validation (SECV), and the residual predictive deviation (RPD) were used to evaluate the calibration models developed [18,20,21,22].

## 3. Results and Discussion

Table 1 reports the descriptive statistics (e.g., average, standard deviation, range, and coefficient of variation) for the measurement of the dry matter, vitamin C, and ellagic acid content in the KP fruit samples used to develop the NIR calibrations. Table 2 shows the cross validation and validation statistics for the prediction of vitamin C and ellagic acid in the set of whole and pureed KP fruit samples analysed. As stated above, the SECV and the RPD (SD/SECV) were used to evaluate the ability of the PLS models developed to predict these parameters [18,20]. SECV is a quantitative measure of how precise the samples are predicted during validation where the bias is a systematic deviation of the predicted values from the true value due to a particular measurement method [18,20]. The SECV and RPD values were 1.81% dry matter (DM) with an RPD of 2.1, and 3.8 mg g^−1^ DM with an RPD of 1.9 for the prediction of vitamin C and ellagic acid, respectively, in the set of whole KP fruit samples. Using the set of pureed KP samples, the SECV and RPD values were 1.73% DM with an RPD of 2.2, and 5.6 mg g^−1^ DM with an RPD of 1.3 for the prediction of vitamin C and ellagic acid, respectively. According to other authors, an RPD value between 2 and 2.5 might indicate that rough quantitative predictions could be possible, while a value between 2.5 and 3 or above might be associated with good and excellent prediction accuracy [18,20,21,22,23]. The RPD values in this study were between 1.3 to 2.2 for the prediction of vitamin C and ellagic acid. Similar SECV values were reported by other authors using mid infrared spectroscopy to predict ellagic acid in coastal oak samples [24].

R^2^ indicates the percentage of variance present in the true component values, which will be reproduced in the prediction (18, 20–23). Depending on the R^2^ values obtained during the calibration process, the NIR models can be classified as follows: possessing a low correlation (0.26 < R^2^ < 0.49), models that can be used to discriminate between a low and high composition of samples (0.50 < R^2^ < 0.64), models that can be used for a rough prediction of the composition (0.65 < R^2^ < 0.81), possessing a good correlation (0.82 < R^2^ < 0.90), and having excellent precision (R^2^ > 0.90) [18,20,21,22,23]. The PLS calibration models developed using the pureed KP samples explained between 48% and 86% of the variation related to vitamin C and ellagic acid, while 55% to 57% of the variation was explained in the calibration models using the whole KP fruit samples. The observed differences in the PLS models were associated with sample presentation (whole vs. pureed fruit). 

It has been reported that the NIR spectra are comprised of wide bands originating from overlapping absorptions corresponding to overtones and combinations of vibrational modes involving C-H, O-H, and N-H chemical bonds [8,25,26]. Although the water absorption bands related to the O-H bonds are predominant in the NIR spectra of fruit such as KP fruit, other molecules can be measured [8,25,26]. Carbohydrates, organic acids, proteins, and other minor compounds can exhibit wide absorption bands as a result of complex hydrogen bonding interactions with different molecules in the NIR wavelength range [8,24,25]. Therefore, the interpretation of the NIR spectra is not as straight forward as the interpretation of the MIR region [8,25,26].

**Figure 1:**
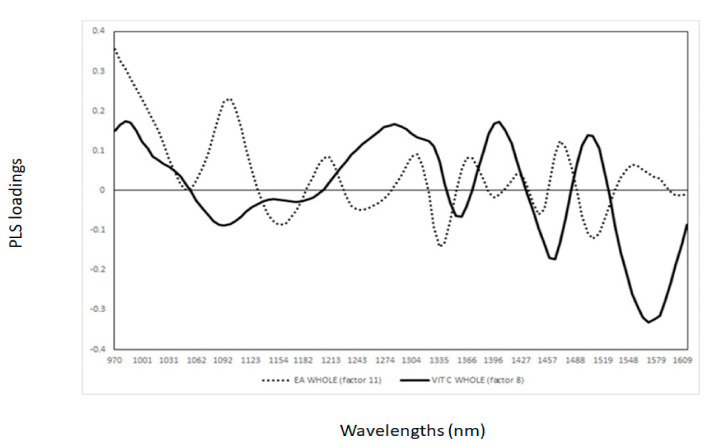
Partial least square loadings for the measurement of vitamin C and ellagic acid in whole Kakadu plum fruit samples.

**Figure 2:**
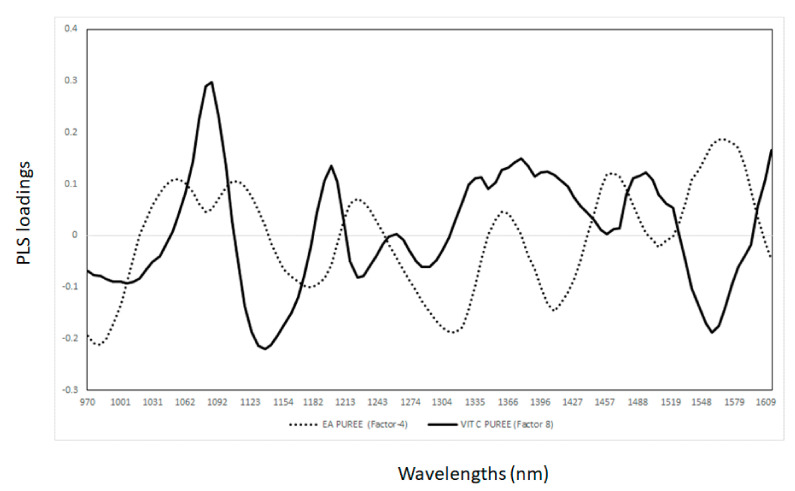
Partial least square loadings for the measurement of vitamin C and ellagic acid in pureed Kakadu plum fruit samples.

In order to understand the basis of the NIR calibrations developed, the PLS loadings were analysed and interpreted for each of the sample presentations used to develop the calibrations for vitamin C and ellagic acid (e.g., whole or pureed fruit; Figure 1 and Figure 2). The relationships between the wavelength and PLS latent variables/loadings imply that these wavelengths contribute to explaining the developed models [18,20,21,22]. Therefore, the value and direction (e.g., positive and negative) of the PLS loading indicated the contributions of individual wavelengths to the model [18,20,21,22]. It has been reported that when PLS models are developed for the same parameters, using different pre-processing or sampling presentation modes for the same sample, they can utilise different wavelengths or loadings. In this study, sample presentations (whole vs. pureed) were shown to have an effect by explaining the observed differences in the PLS calibrations and loadings. The loadings used by the PLS calibrations for the measurement of vitamin C and ellagic acid in the KP puree samples are shown in Figure 1. The loadings for vitamin C were observed at wavelengths of around 1137 nm (C-H combination, aromatic groups), 1217 nm (C-H_2_), 1299 nm (first overtone of C-H combination), 1465 nm (N-H associated with secondary amines), and 1558 nm (O-H), whereas for ellagic acid, the most important wavelengths were observed at 1174 nm (C-H), 1310 nm (first overtone of C-H combination), 1410 nm (O-H bonds), and 1510 nm (N-H amide) [8,25,26,27,28,29,30,31,32,33]. The PLS loadings observed for the calibrations developed using the whole KP fruit samples are shown in Figure 2. The main loadings were observed at 1093 nm (C-H, aromatic groups), 1347 nm (C-H), 1465 nm (N-H), and 1570 nm (N-H) for vitamin C, while for ellagic acid, four wavelengths were observed to influence the models, at 1155 nm (C-H), 1242 nm (C-H), 1440 nm, and 1508 nm (C-H and N-H) [8,25,26,27,28,29,30,31,32,33]. It has been observed that the calibration models for the same parameters used similar wavelengths, and these might indicate that the sample presentation (whole vs. pureed fruit samples) might not have a greater effect on the information collected by the NIR instrument for the prediction of the bioactive compounds in the set of KP fruit samples analysed.

**Figure 3:**
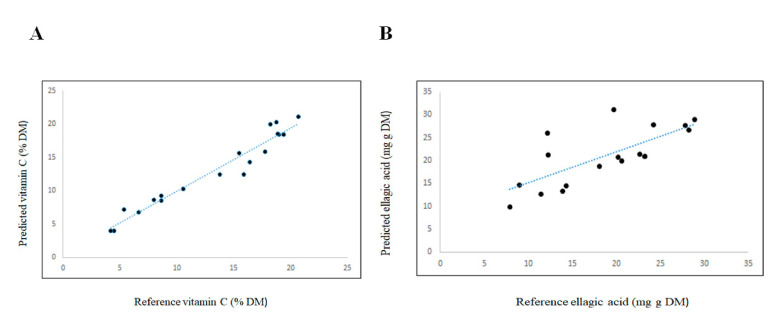
Scatter plot for the validation (*n* = 20) of the measurement of vitamin C (Panel (**A**)) and ellagic acid (Panel (**B**)) in the pureed Kakadu plum samples.

**Figure 4:**
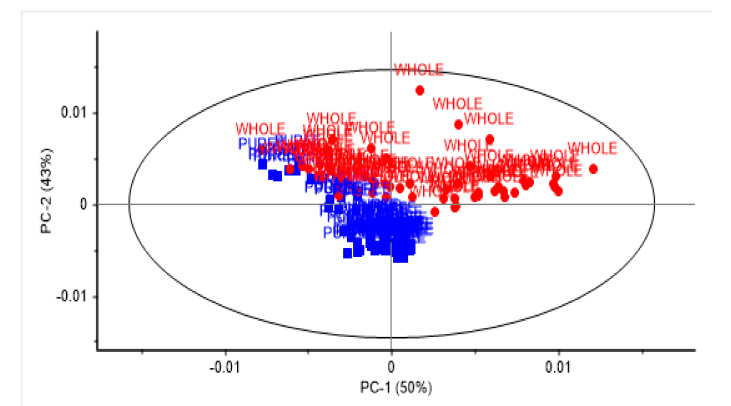
Principal component score plot of Kakadu plum samples analysed as pureed or as whole fruit using near infrared spectroscopy.

Figure 3 shows the scatter plot for the validation of the measurement of vitamin C and ellagic acid in the pureed samples. The influence of the region or origin of the samples was observed upon cross-validation models developed for vitamin C (bimodal distribution as a result of region). However, this trend was not observed for the prediction of ellagic acid in the KP fruit samples analysed. In addition, one and three outlier samples were observed in the prediction of vitamin C and ellagic acid, respectively. A detailed analysis of these outlier samples indicated that they corresponded to spectral outliers. These results are in agreement with those reported by other authors, who indicated that region might have an effect on the concentration of some of these bioactive compounds [1,2,3,7]. It is well known that vitamin C is an important parameter because of its important health and antioxidant properties, which have received a great deal of attention, thus necessitating the development of rapid analytical methods [26]. However, some authors have reported unsatisfactory results using short wavelengths in the NIR region or when the samples contain low concentrations of vitamin C (less than 10 g L^−1^) [22,28,29,30,31,32,33]. Another reported issue might be related to the effect of moisture and its interference when determining the presence of compounds with low concentrations. Recently, Oliveira-Folador and collaborators [33] suggested that the high water content of the pulp of fruit (approximately 84%) contributes to the inherent complexity of NIR spectra. This might also be explained by the fact that the NIR spectral range used is highly sensitive to elements that modify light diffusion, such as physical structure and the presence and content of water in the sample [20,21]. The physical structure of the fruit has been reported to have a large effect on the acquisition of spectra, and this is strongly influenced by the light scattering phenomena, as reported by other authors in different types of fruit and vegetables [22,28,29,30,31,32]. Figure 4 shows the principal component score plot of the KP samples scanned as whole and pureed fruit. Two groups were observed related to the sample presentation used. Whole samples tended to scatter along principal component one (50% of the variation), while most of the pureed samples were clustered together. It is also important to highlight that the NIR spectrum of fresh materials is essentially composed of a large set of overtones and combination bands. This combination, together with the complex chemical composition of a typical fruit or vegetable, makes the near infrared spectrum highly complex [8,25]. Regardless of these issues, the NIR region used in this study showed a high applicability for the rapid screening of samples for high, medium, and low vitamin C and ellagic acid.

## 4. Conclusions

The results of this study showed the ability of a handheld NIR instrument to predict vitamin C and ellagic acid in both whole and pureed KP fruit samples. Although the RPD values obtained are not considered adequate to quantify these bioactive compounds, they can be used to quickly screen the fruit for high- and low-quality vitamin C. The handheld instrument used in this study can be an alternative for rapid and throughput screening of raw materials in remote areas, where it might not be appropriate to use other types of instruments to assess fruit quality (e.g., bioactive compounds). However, further studies are needed to optimize the prediction models for these bioactive compounds and to evaluate the effect of region/origin and harvest (years) in order to make the models more robust for routine applications.

## Figures and Tables

**Table 1:** Descriptive statistics for the measurement of vitamin C and ellagic acid in Kakadu plum fruit samples analysed using NIR spectroscopy.

	% DM	VIT C (% DM)	EA (mg g^−1^ DM)
Average	16.4	12.5	20.64
SD	1.2	3.81	7.7
Minimum	14.2	7.8	7.6
Maximum	18.7	19.3	31.5
CV (%)	7.3	30.4	37.4

CV—coefficient of variation (CV = SD/mean); DM—dry matter; EA—total ellagic acid; SD—standard deviation; VIT C—vitamin C.

**Table 2:** Cross validation and validation statistics for the prediction of ellagic acid and vitamin C in whole and pureed Kakadu plum sample analyses using near infrared reflectance spectroscopy.

		R^2^_CV_	SECV	Slope	Bias	LV	RPD_CV_	r	SEP
Whole	VIT C (% DM)	0.55	1.81	0.53	0.029	8	2.1	0.85	2.0
	EA (mg g^−1^ DM)	0.57	3.8	0.61	−0.007	11	1.96	0.55	7.5
									
Puree	VIT C (% DM)	0.86	1.73	0.87	0.10	8	2.2	0.89	1.9
	EA (mg g^−1^ DM)	0.48	5.6	0.57	0.002	11	1.3	0.56	6.2

CV—cross validation; DM—dry matter; LV—number of optimal latent variables used to develop the models; VIT C—vitamin C; EA—total ellagic acid; R^2^CV—coefficient of determination in cross validation; r—correlation coefficient in prediction; RPD—SD/SECV; SECV—standard error for cross validation; SEP—standard error of prediction.
